# Preventive effect of oral goshajinkigan on chronic oxaliplatin-induced hypoesthesia in rats

**DOI:** 10.1038/srep16078

**Published:** 2015-11-06

**Authors:** Toru Kono, Yasuyuki Suzuki, Keita Mizuno, Chika Miyagi, Yuji Omiya, Hitomi Sekine, Yasuharu Mizuhara, Kanako Miyano, Yoshio Kase, Yasuhito Uezono

**Affiliations:** 1 Faculty of Pharmaceutical Sciences, Hokkaido University, W-6, N-12, Kita-ku, Sapporo 060-0812, Japan; 2 Center for Clinical and Biomedical Research, Sapporo Higashi Tokushukai Hospital, 3-1, N-33, E-14, Higashi-ku, Sapporo 065-0033, Japan; 3 Tsumura Research Laboratories, Kampo Scientific Strategies Division, Tsumura & Co., 3586 Yoshiwara, Ami-machi, Inashiki-gun, Ibaraki 300-1192, Japan; 4 Division of Cancer Pathophysiology, National Cancer Center Research Institute, 5-1-1 Tsukiji, Chuo-ku, Tokyo 104-0045, Japan

## Abstract

Oxaliplatin, a widely used chemotherapeutic agent, induces peripheral neuropathy that manifests itself as two distinct phases: acute cold hyperesthesia and chronic peripheral hypoesthesia/dysesthesia. The latter is a serious dose-limiting side effect that can often lead to withdrawal of treatment. We have developed a rat model expressing both phases and used the model to investigate the action of goshajinkigan (GJG), a traditional Japanese herbal medicine, which was reported to ameliorate oxaliplatin-induced neuropathy in a placebo-controlled double-blind randomized phase II study. In this study, neuropathy was induced by injection of oxaliplatin twice weekly for 8 wks. The maximum level of cold hyperesthesia was observed at 4 wks with heat hypoesthesia developing later. Microscopy studies revealed atrophy of axons of myelinated sciatic nerve fibers in oxaliplatin-treated rats at 8 wks. Co-administration of GJG ameliorated both abnormal sensations as well as histological damage to the sciatic nerve. A pharmacokinetic study revealed numerous neuroprotective components of GJG that are rapidly absorbed into the blood. GJG and some of its components attenuated the generation of oxaliplatin-induced reactive oxygen species, which is a possible mechanism of oxaliplatin-induced neurotoxicity. The present study provides a useful animal model for oxaliplatin-induced neurotoxicity as well as a promising prophylactic agent.

Peripheral neuropathy is a well known frequent adverse effect of oxaliplatin treatment^1^,^2^,^3^,^4^,^5^. Acute peripheral neuropathy is characterized by hyperesthesia to cold stimulation and temporarily reduces the patient’s quality of life (QOL)^6^,^7^. Chronic peripheral neuropathy is characterized by hypoesthesia and dysesthesia as characteristic chronic oxaliplatin-induced neuropathic symptoms^8^,^9^ and worsens the QOL over the long term. Chronic peripheral neuropathy is the most common dose-limiting factor, so prevention and amelioration of these symptoms is very important for continuous oxaliplatin chemotherapy.

Animal studies have confirmed that treatment with oxaliplatin for a short period of time often results in hyperesthesia to cold stimulation^10^,^11^,^12^,^13^,^14^,^15^. However, there are no reports of reproduction of the abnormal sensations (especially hypoesthesia) in an animal model. Some studies showed hypoesthesia to heat stimulation in animal models with diabetic neuropathy^16^,^17^. Therefore, perception of heat may be a useful method to evaluate abnormal sensations resulting from oxaliplatin-induced chronic neuropathy. A previous report observed that peripheral blood flow was also altered in oxaliplatin-treated animals, which might correlate with neurotoxicity^18^. In addition, several studies have suggested that increased production of reactive oxygen species (ROS) due to treatment with oxaliplatin can lead to the onset of neurotoxicity^11^,^19^.

Goshajinkigan (GJG), a traditional Japanese herbal medicine, has been widely used to treat disease-associated neuropathy (i.e., diabetic neuropathy)^20^,^21^,^22^. Recently, the preventive effect of GJG against oxaliplatin-induced peripheral neurotoxicity in a placebo-controlled double-blind randomized phase II study (the GONE Study) was reported^23^. Animal investigations also demonstrated that GJG suppresses oxaliplatin-induced cold hyperalgesia^10^,^24^. However, all of these studies were conducted using acute rather than chronic models.

In the present study, we first examined the effect of oxaliplatin-treatment for a period of 8 wks on the perception of heat and cold stimulation as well as on histopathology and peripheral blood flow using rats. Second, we evaluated the effects of GJG on oxaliplatin-induced chronic neurotoxicity. Third, pharmacokinetic studies of GJG and the effects of GJG and its components on oxaliplatin-induced ROS generation were performed to explore the mechanism of the observed beneficial effect of GJG.

## Results

### GJG ameliorated cold hyperesthesia and heat hypoesthesia induced by oxaliplatin

**Figure 1:**
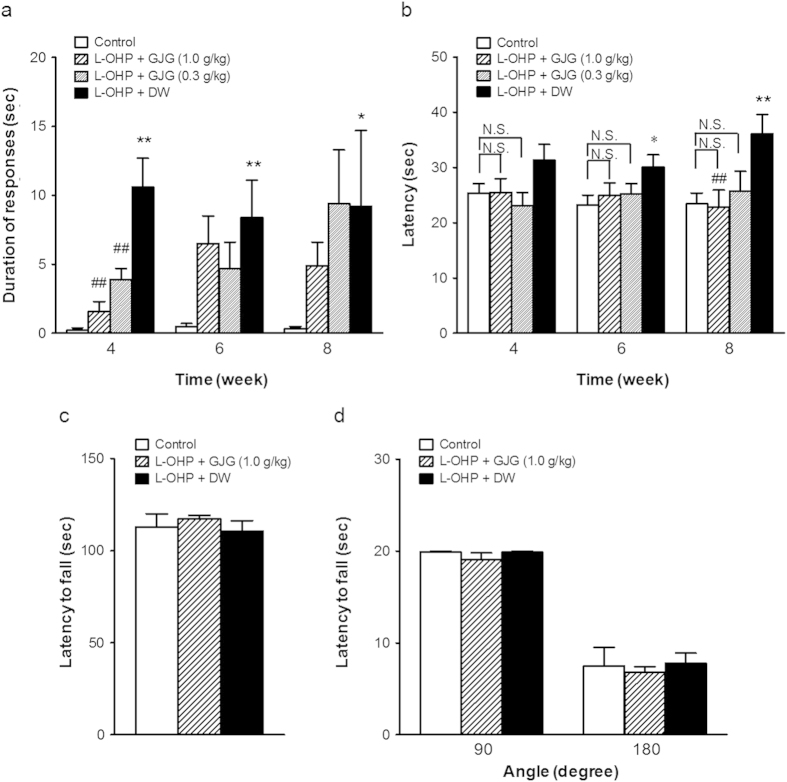
Effect of goshajinkigan (GJG) on oxaliplatin-induced cold hyperalgesia, heat hypoalgesia and motor function in rats. Oxaliplatin (L-OHP, 4 mg/kg) or its vehicle (control) was administrated intraperitoneally twice a week for 8 wks. GJG (0.3, 1.0 g/kg) or distilled water (DW) was simultaneously and orally administered. The acetone test (**a**) for assessment of cold hyperalgesia or hot plate test (**b**) for assessment of heat hypoalgesia induced by oxaliplatin was performed at 4, 6 and 8 wks. Data are expressed as mean ± S.E.M.; N = 6–10 per group. *P < 0.05, **P < 0.01 compared with control group. ^#^P < 0.05, ^##^P < 0.01 compared with L-OHP + DW. N.S.: no significant difference. The rotarod test (**c**) or wire hang test (**d**) was performed at 8 wks. GJG (1.0 g/kg) or DW was simultaneously and orally administered at oxaliplatin-treatment. Data are expressed as mean ± S.E.M.; N = 8–12 per group.

Treatment with oxaliplatin (4 mg/kg, intraperitoneally, twice weekly) significantly increased the duration of withdrawal responses to cold stimulation by acetone at 4, 6, and 8 wks, compared to the naïve control. GJG (0.3 or 1.0 g/kg, p.o.) prevented cold hyperalgesia induced by oxaliplatin at 4 wks but not 6 and 8 wks (Fig. 1a). In the hot plate test (Fig. 1b), there was no significant difference in the latency of withdrawal to heat stimulation among all the groups at 4 wks after oxaliplatin treatment. Thereafter, that in the oxaliplatin-treated group compared to the naïve control gradually increased. A statistically significant difference was obtained at 6 wks and 8 wks, at the level of p < 0.05 and <0.01, respectively. These data suggest that cold hyperalgesia is rapidly induced and maintained, while heat hypoesthesia develops more slowly. The groups co-administered with GJG at the dose of 0.3 or 1.0 g/kg (p.o.) gave no significant change in the withdrawal responses to heat stimulation compared with the naïve control groups throughout the experimental periods. In particular, at 8 wks, the withdrawal responses to heat stimulation of 1.0 g/kg of GJG-treated group were significantly lower than those of the oxaliplatin-treated group. Next, we examined whether motor impairment is involved in the effects induced by oxaliplatin and/or GJG treatment. The effect of oxaliplatin on motor coordination was examined by a rotarod test. After treatment with oxaliplatin for 8 wks, there were no significant differences in motor coordination among the tested groups (Fig. 1c). In the wire screen hanging test of muscle strength, no significant differences in the performances of the different groups of rats were observed when the wire screen was inclined to either 90° or 180° for 20 seconds (Fig. 1d).

### GJG prevented oxaliplatin-induced damage of myelinated axons in the sciatic nerve

**Figure 2:**
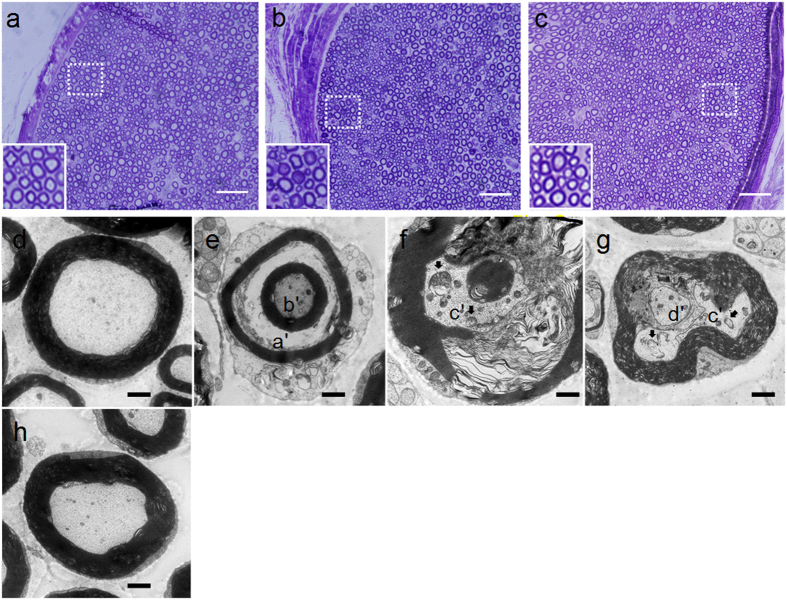
Oxaliplatin (L-OHP)-induced axonal degeneration in rat sciatic nerve and goshajinkigan (GJG)-ameliorated axonal degeneration of the sciatic nerve. Histological analysis of the sciatic nerve was performed at 8 wks. (**a**–**c**) Representative cross sections of the sciatic nerve by light microscopy. The sciatic nerves were isolated in vehicle-treated (**a**), L-OHP + DW-treated (**b**) and L-OHP + GJG (1 g/kg)-treated rat (**c**), and then stained with toluidine blue. Scale bar = 100 μm. (**d**–**h**) Representative pictures of the sciatic nerve by electron microscopy. (**d**) Axons of myelinated fiber in vehicle-treated rat. (**e**) Myelin sheath (a’) by atrophy of axon (b’) in L-OHP + DW-treated rat. (**f**) Accumulation of organelles (c’), vacuolated and swollen mitochondria (arrows) in degenerative axon of L-OHP + DW-treated rat. (**g**) Infiltration of macrophage (d’) in myelin sheath and phagocytosis of degenerative axon in L-OHP + DW-treated rat. (**h**) A representative cross section of axon treated with GJG. Scale bar = 10 μm.

In vehicle-treated rats, no histological changes to sciatic nerve were observed by light and electron microscopy at 8 wks (Fig. 2a,d). Degenerated axons in the sciatic nerves of oxaliplatin-treated rats were observed by light microscopy (Fig. 2b). Examination by electron microscopy showed that oxaliplatin caused atrophy of axons of myelinated fibers in the sciatic nerve (Fig. 2e). There were no notable morphological changes of non-myelinated nerve fibers in the sciatic nerves between the control and oxaliplatin groups (data not shown). According to our electron microscopic analysis at low magnification, the ratio of degenerated axons in total myelinated nerve fibers in the oxaliplatin-treated group was higher than that in the control group (Control, 1.0% (n = 2); oxaliplatin, 8.8 ± 2.4% (n = 3)). Degenerated axons of oxaliplatin-treated rats displayed the accumulation of organelles, vacuolated and swollen mitochondria, and infiltration of macrophages (Fig. 2f,g). The frequency of these histological changes was decreased by 1 g/kg GJG treatment (Fig. 2c,h). (Oxaliplatin + GJG, 3.5 ± 1.5% (n = 3)).

### GJG ameliorated the oxaliplatin-induced decrease in peripheral blood flow

**Figure 3:**
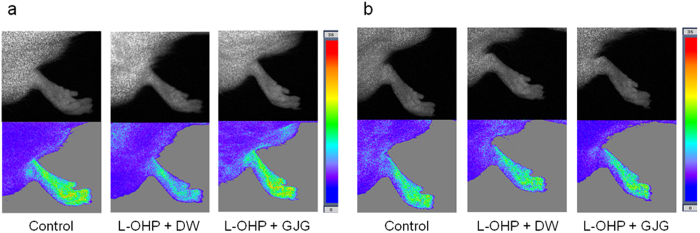
Alteration of mean blood flow in left hindlimb in oxaliplatin (L-OHP)-treated rats at 4 (a) and 8 (b) wks. Real and color images of blood flow on a two-dimensional laser speckle Blood Flow Imager. Blood flow values analyzed by region of interest in the left hindlimb.

Regional blood flow, except for that in the ears and tail, decreased from 10 days to 4 wks after treatment with oxaliplatin, but then increased back toward normal values after 8 wks (Table 1). Mean blood flow in the fore- and hindlimbs was significantly decreased at 4 wks. Reduction of mean blood flow in the left hindlimb was significantly inhibited in rats administered GJG (1 g/kg) at 4 wks after oxaliplatin treatment (Fig. 3a). No change in blood flow at each site was observed at 8 wks after oxaliplatin treatment (Fig. 3b). GJG did not affect either systemic blood pressure or heart rate in oxaliplatin-treated rats (data not shown).

### Pharmacokinetic study of GJG-derived components in rat plasma

**Figure 4:**
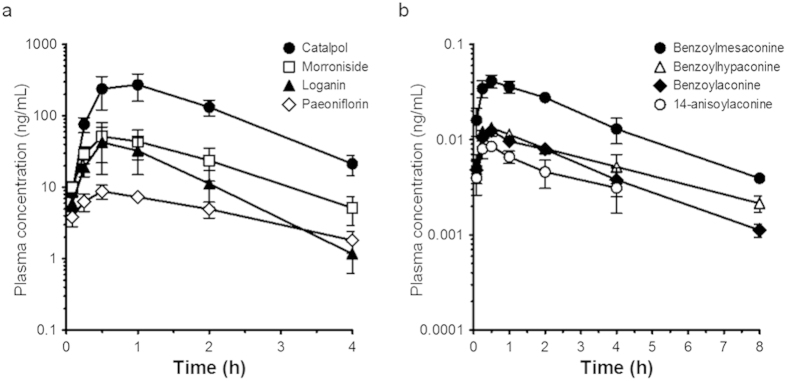
Plasma concentration-time curves of goshajinkigan (GJG)-derived compounds after oral administration to rats. The concentrations of loganin, morroniside, catalpol, paeoniflorin (**a**) and benzoylaconine, benzoylmesaconine, benzoylhypaconine, 14-anisoylaconine (**b**) in plasma were detected by LC-MS/MS. Data are expressed as mean ± S.D.; N = 4 per test compound in group (**a**), N = 6 per test compound in group (**b**).

GJG-derived compounds were measured under two different analytical conditions. Iridoid glucosides, loganin (LO), morroniside (MO), catalpol (CA), and a monoterpene glucoside, paeoniflorin (PF), were detected in plasma after oral administration of GJG (1.0 g/kg) (Fig. 4a). Figure 4b shows the plasma concentrations of benzoylmesaconine, benzoylhypaconine, benzoylaconine and 14-anisoylaconine (14-AA). LO and MO, components of *Corni* fructus, PF, a component of *Moutan* cortex, and benzoylaconines, constituents of processed *Aconiti tuber*, were absorbed quickly and reached the maximum plasma concentration (C_max_) at 30 minutes; CA, a component of *Rehmanniae* radix reached C_max_ at 60 minutes after oral administration of GJG (Table 2).

### GJG-derived compounds inhibited ROS generation caused by oxaliplatin in neuro2A cells

**Figure 5:**
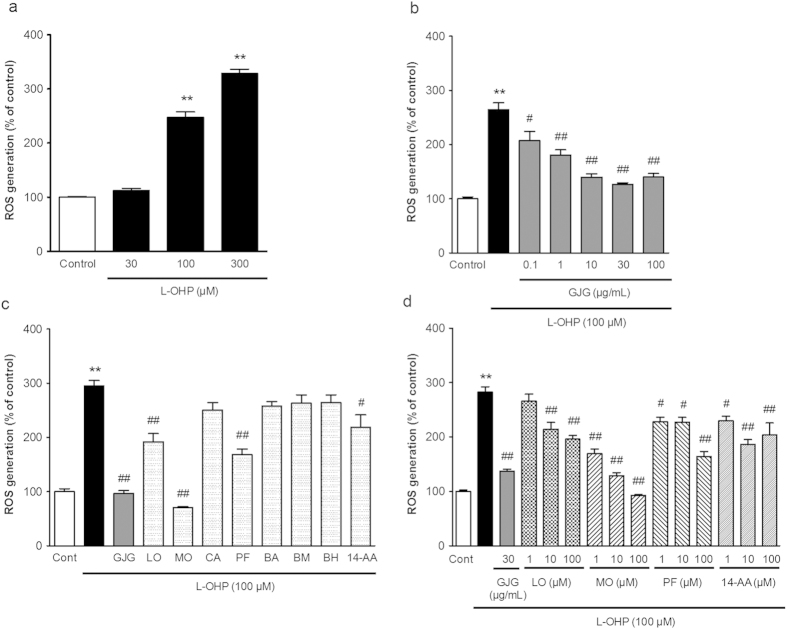
Attenuation of oxaliplatin-induced ROS generation in neuro2A cells by goshajinkigan (GJG)-derived constituents. Neuro2A cells were seeded at 2.0 × 10^4^ cells/well in 96-well plates. After treatment with drugs, ROS generation was measured using CM-H_2_DCFDA. (**a**) Neuro2A cells were treated with oxaliplatin (L-OHP; 30–300 μM) for 1 h, and ROS production was measured. (**b**) GJG (0.1–100 μg/mL) was simultaneously administered with 100 μM L-OHP for 1 h. (**c**) First screening for active components of GJG. GJG (30 μg/mL) and 8 eight compounds (loganin: LO, morroniside: MO, catalpol: CA, paeoniflorin: PF, benzoylaconine: BA, benzoylmesaconine: BM, benzoylhypaconine: BH, 14-anisoylaconine: 14-AA, 100 μM each) of GJG detected in rat plasma after oral administration of GJG were simultaneously administered with L-OHP, respectively. (**d**) Second screening for active components of GJG. GJG (30 μg/mL), loganin (LO), morroniside (MO), paeoniflorin (PF) and 14-anisoylaconine (14-AA) (1–100 μM) were applied with L-OHP for 1 h, respectively. Data are expressed as mean ± S.E.M. **P < 0.01 vs. control. ^#^P < 0.05, ^##^P < 0.01 vs. L-OHP alone.

Exposure to oxaliplatin (30–300 μM) for 1 hour increased ROS generation in neuro2A cells (Fig. 5a), which are inhibited by two representative antioxidants; glutathione and N-acetyl cysteine (Supplementary Figure S1). GJG (0.1–100 μg/mL), administered simultaneously with 100 μM oxaliplatin, attenuated the oxaliplatin-induced generation of ROS in a concentration-dependent manner (Fig. 5b). Next, we attempted to clarify the active components in GJG. Thus, we examined the effect of eight compounds derived from GJG on oxaliplatin-induced ROS generation. Co-administration of LO, MO, PF or 14-AA with oxaliplatin significantly decreased ROS generation (Fig. 5c,d).

### GJG did not affect platinum concentration in dorsal root ganglia (DRG).

**Figure 6:**
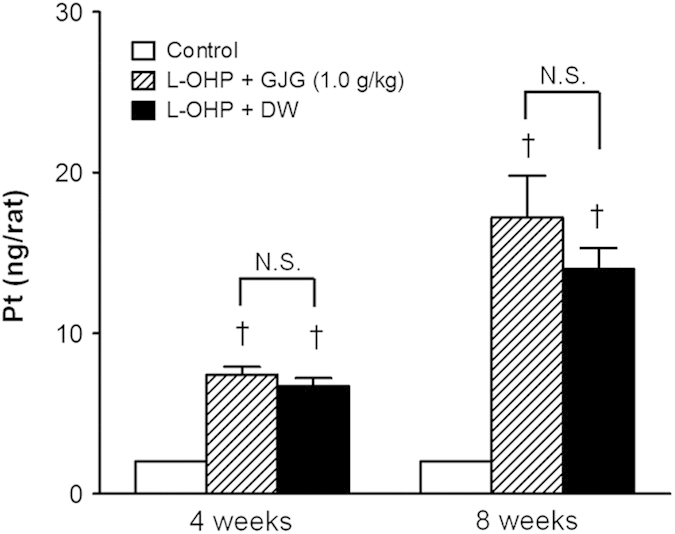
Accumulation of platinum in dorsal root ganglia (DRG) of oxaliplatin (L-OHP)-treated rat and the effect of goshajinkigan (GJG) administration. DRG at lumbar L4–6 were isolated from each group at 4 and 8 wks after L-OHP-treatment. L-OHP (4 mg/kg) or its vehicle (control) was administrated intraperitoneally twice a week for 8 wks. DRG platinum levels were determined by an ICP-MS system. GJG (1.0 g/kg) or distilled water (DW) was immediately injected orally after administration of L-OHP. Results are expressed as mean ± S.E.M.; N = 6–10 per group. *P < 0.01 compared with control group. N.S.: no significant difference.

The increase in platinum accumulation in the rat DRG directly correlated with the duration of oxaliplatin treatment. GJG did not affect the accumulation of platinum associated with oxaliplatin treatment (Fig. 6).

## Discussion

The present model provides four altered phenotypes that may be related to oxaliplatin-induced side effects. Cold hyperesthesia was established after 4 wks of oxaliplatin treatment and persisted at least until the end of the study period (i.e., 8 wks). By contrast, the onset of heat hypoesthesia occurred more slowly, with a significant alteration in heat sensitivity evident only after 6 wks, and still developing at 8 wks. The damage to myelinated axons in the sciatic nerve was clearly observed at 8 wks. Peripheral blood flow decreased at 4 wks but thereafter returned to normal levels. Although GJG showed ameliorating effects for all these symptoms, we believe these side-effects may occur *via* different mechanisms.

In clinical practice, acute oxaliplatin-induced hypersensitivity to cold stimuli is generally observed shortly after the commencement of oxaliplatin therapy. Several reports^14^,^25^,^26^ suggest enhanced response of transient receptor potential ankyrin 1 (TRPA1), a polymodal ion channel sensitive to cold temperature and chemical stimuli, is involved in the pathogenesis of oxaliplatin-induced acute cold hyperalgesia and that therapies targeting transient receptor potential (TRP) channels may be beneficial. We^10^ and Kato *et al.*^27^ have reported the possibility that GJG ameliorates cold hyperalgesia *via* modulation of function and expression, respectively, of TRP channels.

Compared to the rapid expression of cold hyperesthesia, there is a delay in the development of peripheral sensory neuropathy such as pain, numbness and tingling (typically in the hands and feet)^8^,^9^. The latter neuropathy, including heat and mechanical hypoesthesia/dysesthesia, is known to be closely related to the cumulative dose of oxaliplatin^28^. The neuropathy is thought to be related to damage in the peripheral sensory nerves^29^,^30^ which, in the present study, is clearly evident by histological changes to the sciatic nerve. The suppression, or at least retardation, in the development of sensory nerve damage by GJG may contribute to the amelioration of heat hypoesthesia. Thus, direct protection of neuronal cells by components of GJG, rather than modulation of the TRPA1 channel, could to be involved in the amelioration of the later phase neuropathy. Although chronic peripheral neuropathy is the most serious problem associated with oxaliplatin therapy, there is no available appropriate animal model to examine oxaliplatin-induced hypoesthesia. Therefore, the present model, showing both heat hypoesthesia and cold hyperesthesia with preserved motor function, may be a good model of human symptoms caused by long-term oxaliplatin administration^9^.

Despite the number of studies on oxaliplatin-induced neuropathy, there are relatively few that have focused on the derangement of peripheral blood flow. Although a possible relationship between mechanical allodynia and decreased blood flow has been suggested^18^, the significance and pathophysiology of blood flow disorder in oxaliplatin-treated patients remains unclear. GJG and its components have been reported to show vasodilatation in several models partly *via* increasing nitric oxide production^31^,^32^,^33^,^34^. A similar mechanism may be involved in the vasodilatation by GJG observed in the present experiments. However, a future extensive study to clarify the mechanisms and significance of the effect is needed.

Light and electron microscopy studies revealed scattered axonal damage in sciatic nerves of the oxaliplatin group. Our observations suggested that oxaliplatin induces axonal degeneration in the sciatic nerve. Indeed, these structural changes are consistent with a previous study^29^,^30^. The morphological damage to mitochondria induced by oxaliplatin may result in mitochondrial dysfunction, which could increase oxidative stress in peripheral nerve tissue during chemotherapy. Recent studies demonstrated that treatment with oxaliplatin increased ROS production and induced lipid peroxidation and protein and DNA oxidation in neuronal cells and peripheral nerve tissue^11^,^19^.

Furthermore, the administration of several compounds with antioxidant properties, such as curcumin^35^ and quercetin^36^, improved oxaliplatin-induced neuropathy. These reports suggested that oxidative stress is responsible for oxaliplatin-induced neuropathy. Thus, oxidative stress could play an important role in our chronic neuropathy model, and treatment with antioxidants may be useful in preventing oxaliplatin-induced neurotoxicity.

In the pharmacokinetic study of GJG, we confirmed that eight components of GJG were rapidly absorbed, some of them at high concentrations, in rat plasma after oral administration of GJG. Some of these components are thought to possess neuroprotective and antiradical activities. MO, LO and PF have been reported to attenuate ROS production, decrease mitochondrial membrane potential and inhibit apoptosis induced by H_2_O_2_ or amyloid β 25–35 peptide in SHSY-5Y cells^37^,^38^,^39^. CA and MO have been reported to have neuroprotective activity by suppressing ROS generation in other experimental systems^40^,^41^,^42^. Furthermore, direct ROS scavenging action was observed for PF using electron spin resonance assay^43^. Therefore, we investigated whether these eight compounds inhibit intracellular ROS generation induced by oxaliplatin using mouse neuroblastoma neuro2A cells. As shown in Fig. 5a, dichloro (1,2-diaminocyclohexane) platinum (Pt(dach)Cl_2_, data not shown), an active metabolite of oxaliplatin, increased ROS generation in neuro2A cells, but oxalate, another metabolite of oxaliplatin, did not (data not shown). These results suggest that oxaliplatin increases ROS generation due to Pt(dach)Cl_2_ but not oxalate. Among the tested components, LO, MO, PF and 14-AA, as well as GJG, significantly reduced ROS generation. In particular, MO showed the strongest action among these active components. The results suggest that these active components may be involved in the preventive effects of GJG on oxaliplatin-induced neurotoxicity *via* suppression of ROS generation. However, these four compounds alone may not be sufficient to fully explain the potent anti-ROS activity of GJG because their respective concentrations in 30 μg/ml GJG will be far less than 10 μM (i.e., around 0.3 μM (MO); for more detail, see Supplementary method). It is plausible that GJG contains additional untested and/or unidentified antiradical components. Alternatively, the combination of various components of GJG might exert a synergistic effect beyond the simple sum of the activities of each individual compound.

MO, LO and CA reached a maximum plasma concentration between 30 and 60 minutes after oral administration of GJG and the high concentration was then maintained for 4 hours. In a previous report, rats injected with 5 mg/kg of oxaliplatin intraperitoneally showed a maximum plasma concentration of platinum approximately 90 minutes after administration, which was maintained for 2 hours^44^. Oral administration of GJG with simultaneous intraperitoneal injection of oxaliplatin showed a preventive effect for oxaliplatin neuropathy in rats. This preventive effect of GJG could be explained by absorption of the neuroprotective components found in GJG that reached the DRG before oxalate and Pt(dach)Cl_2_ 8,45. Oxaliplatin-induced chronic neuropathy may be caused by accumulation of platinum and active metabolites in neuronal cells^46^. Taken together, these lines of evidence indicate that GJG should be administered orally before oxaliplatin in order to efficiently exert the preventive effect of GJG.

We have conducted Phase II and III trials to investigate whether GJG ameliorates the adverse effects of oxaliplatin-induced neuropathy. The Phase II trial showed GJG exhibited a promising effect on the neuropathy but the Phase III trial did not. It is obviously important to understand why such different outcomes were obtained from the two trials. The present results may help to shed some light on this apparent discrepancy. Specifically, the treatment protocols in the clinical studies did not consider the pharmacokinetic data of the GJG components. Thus, these trials may not have taken into account the most effective timing for GJG administration. In particular, the Phase III trial was a multicenter study in which the timing of GJG administration was not strictly controlled due to the lack of detail in the protocol. However, a human pharmacokinetic study of GJG is scheduled to begin shortly, which will be followed by a new clinical study.

Oxaliplatin produces antitumor effects by platinum accumulation in tumor cells. Clearly, oxaliplatin-induced chronic neuropathy symptoms must be ameliorated without affecting its antitumor action, even though chronic neuropathy is a serious clinical problem. In reports on clinical or basic research, GJG did not affect the antitumor efficacy of oxaliplatin^23^,^24^,^47^,^48^. Indeed, the present results indicate that GJG does not alter the accumulation of platinum in rat DRG. Thus, our findings suggest neuroprotection by GJG is not due to the inhibition of platinum accumulation.

In conclusion, we have developed a quantitatively evaluable chronic model of hypoesthesia occurring in rats treated long-term with oxaliplatin. The oral administration of GJG prevented functional and histological disorders of chronic oxaliplatin-induced neurotoxicity without inhibiting the accumulation of platinum. A pharmacokinetic study showed that several neuroprotective compounds were detected at high levels in rat plasma shortly after the oral administration of GJG. In particular, four compounds of GJG, including MO and LO, inhibited oxaliplatin-induced ROS production in neuro2A cells. The present study provides a useful model for the elucidation of oxaliplatin-induced chronic neuropathy and affords some insights on the mechanism of action of GJG.

## Methods

### Animals

Male Sprague-Dawley rats weighing 200–400 g (Japan SLC, Shizuoka, Japan) were kept at a temperature of 23 ± 3 °C, relative humidity of 50 ± 20%, and a 12-hour light/dark cycle. Animals were allowed free access to solid food and water in their home cages. All experimental procedures were ethically approved by the Laboratory Animal Committee of Tsumura and Co. and performed according to the institutional guidelines for the care and use of laboratory animals, which is in accordance with the National Institutes of Health Guide for the Care and Use of Laboratory Animals.

### Drugs

Oxaliplatin was purchased from Wako Pure Chemical Industries, Ltd. (Osaka, Japan), and 5-(and-6)-chloromethyl-2,7-dichlorodihydrofluorescein diacetate acetyl ester (CM-H_2_DCFDA) was purchased from Life Technologies (Carlsbad, CA, USA). Goshajinkigan (GJG; TJ-107) is composed of 10 herbal medicines in fixed proportions: *Rehmanniae* radix 5.0 g, *Achyranthis* radix 3.0 g, *Corni* fructus 3.0 g, *Moutan* cortex 3.0 g, *Alismatics* rhizome 3.0 g, *Dioscoreae* rhizome 3.0 g, *Plantaginis* semen 3.0 g, *Hoelen* 3.0 g, processed *Aconiti* tuber 1.0 g, and *Cinnamomi* cortex 1.0 g. The GJG was prepared as a spray-dried powder from a hot-water extract (Tsumura & Co., Tokyo, Japan). Benzoylaconine, benzoylmesaconine, benzoylhypaconine, 14-anisoylaconine, loganin, morroniside, catalpol and paeoniflorin were obtained from Tsumura & Co. Oxaliplatin was dissolved in 5% glucose solution. GJG was dissolved in distilled water (DW).

### Drug administration

To establish the oxaliplatin-induced chronic neuropathy rat model, oxaliplatin (4 mg/mL/kg) or its vehicle (5% glucose solution) was injected intraperitoneally twice weekly for 8 wks. Rats were orally administered GJG (0.3 or 1.0 g/10 mL/kg) or DW 5 times a week for 8 wks.

### Assessment of thermal sensation

Cold hyperalgesia induced by oxaliplatin was assessed by an acetone test^10^. The acetone test was done on days 24, 38 and 52. Briefly, 250 μL of acetone (Wako Pure Chemical Ltd., Osaka, Japan) was sprayed onto the plantar skin of the right hind paw, and the time spent in elevation and licking of the stimulated hind paw for 60 seconds was measured. Acetone was applied twice at a 15-minute interval, and the average withdrawal response time was calculated. A hot plate test was performed for assessment of thermal hyperalgesia on days 25, 39 and 54. Rats were placed in a Hot/Cold Plate Analgesia Meter (MK-350HC; Muromachi Kikai Co., Ltd., Tokyo, Japan) that kept the temperature of the plate at 50 °C. A cut-off time of 45 seconds was set to prevent tissue damage. The latency of withdrawal response, such as licking and flinching of each hind paw, was recorded.

### Assessment of motor function

A rotarod test was carried out on day 57 to evaluate if chronic treatment of oxaliplatin had any effect on motor coordination. Rats were placed on the rotarod (SN-497; Shinano-Seisakusho Ltd., Tokyo, Japan), which is a horizontal rod 10 cm diameter, with their heads directed against the direction of rotation at 5 rpm. The time that the animal stayed on the rod (up to maximum of 120 seconds) was recorded. A wire hang test was performed for assessment of neuromuscular strength on day 53. Animals were placed on the wire net that was held horizontally. The wire screen was first inclined at 90 ° for 20 seconds, then turned to 180 ° for a further 20 seconds. The latency to fall from the wire net inclined at the respective angles was recorded.

### Histological analysis of the sciatic nerve

The rats were deeply anesthetized with pentobarbital (50 mg/kg), and transcardially perfused with phosphate-buffered saline (pH 7.4), followed by 2.5% phosphate-buffered glutaraldehyde. The sciatic nerves were rapidly dissected, and the samples were kept overnight in the same fixative at 4 °C. The fixed fibers were post-fixed with 1% osmium tetroxide solution for 3 hours, dehydrated in a graded alcohol series, and embedded in Epon815. For light microscopy, semi-thin sections were cut from each block and stained with toluidine blue. The stained sections were observed using a light microscope (BX60, Olympus Corp., Tokyo, Japan). For electron microscopy, ultra-thin sections were cut and stained with uranyl acetate and lead citrate. The stained sections were observed under an electron microscope (model H-7650; Hitachi High-Technologies Corp., Tokyo, Japan). To calculate the ratio of degenerated myelinated nerve fibers, electron micrographs were taken at low magnification (×200). The numbers of normal- and degenerated-myelinated nerve fibers were counted in the area of 0.05 mm^2^ (0.01 mm^2^ × 5 areas), and the ratio of degenerated nerve fibers was calculated as the percentage of total nerve fibers in each group. In this study, the pathological experts who investigate myelination or demyelination analyzed light and electron microscopy in detail.

### Measurement of peripheral blood flow

Peripheral blood flow was measured using a two-dimensional laser speckle Blood Flow Imager (Omegazone OZ-2; Omegawave, Inc., Tokyo, Japan). Rats were anesthetized with isoflurane for induction and urethane intraperitoneally for maintenance. Body temperature was maintained at 37.5 ± 0.5 °C using a heating pad. Blood pressure and heart rate were concurrently measured by a tail-cuff apparatus (BP-98A-L; Softron, Tokyo, Japan). Peripheral blood flow in the head, ears, forelimbs, hindlimbs and tail of rats was measured at four defined time points (day 3, 10, 26 and 54). Values were analyzed by analysis software (LIA-V210, Softron). Real image and color-coded images were obtained in high-resolution mode (639 × 480 pixels; 2 images/second). The blood flow value in each peripheral site is expressed as mean blood flow in the region of interest (ROI). The ratio of blood flow rate of treated animals to naïve animals was calculated (%).

### Pharmacokinetic study of GJG-derived compounds in plasma

Rats cannulated in the jugular vein were fasted for more than 16 hours with access to water. At the time of 0.083, 0.25, 0.5, 1, 2, 4 and 8 hours after oral administration of GJG (1.0 g/10 mL/kg), blood samples were collected with a heparin-coated syringe and centrifuged at 3000 rpm for 15 minutes at 4 °C. The plasma samples were pretreated by precipitating plasma protein with an organic solvent or by solid-phase extraction and injected into the LC-MS/MS system for analysis. Components of GJG in plasma were measured by a QTRAP®5500 system (AB SCIEX, Tokyo, Japan) equipped with an Agilent 1260 HPLC system (binary pump, online degasser, auto plate-sampler and column oven; Agilent Technologies, Tokyo, Japan). The lower limits for quantification were 0.001 ng/mL for benzoylaconine, benzoylmesaconine, benzoylhypaconine; 0.002 ng/mL for 14-anisoylaconine; 0.2 ng/mL for loganin and morroniside; 0.5 ng/mL for paeoniflorin; and 5 ng/mL for catalpol. C_max_ and time to reach maximum plasma concentration (T_max_) as pharmacokinetic parameters were calculated based on the plasma concentration of each compound by Phoenix WinNonlin (version 6.3; Certara L.P., St. Louis, MO, USA).

### Measurement of platinum concentration in dorsal root ganglia (DRG)

Lumbar L4-L6 DRG were isolated from each group on days 26 and 54 after oxaliplatin treatment. The tissue samples were pretreated by heating in an acid solution, and the tissue platinum concentration was analyzed using an ICP-MS system (Agilent 7700s; Agilent Technologies, Inc., Santa Clara, CA, USA). The lower limit for quantification of platinum in tissue was 2.0 ng.

### Evaluation of the anti-oxidative capacity of GJG-derived compounds

Mouse neuroblastoma Neuro2A cells (ATCC-CCL 131) were grown in Dulbecco’s MEM (Life Technologies) containing 10% fetal bovine serum with 100 units/mL penicillin and 100 mg/mL streptomycin (Life Technologies). The cells were maintained at 37 °C in a humidified atmosphere with 5% CO_2_ and 95% air. For measurement of ROS generation, the cells were seeded at 2.0 × 10^4^ cells/well in poly-D-lysine-coated 96-well plates (BD Falcon, Franklin Lakes, NJ, USA). After plating, the cells were maintained in Dulbecco’s MEM (without phenol red or serum, Life Technologies) with 100 units/mL penicillin and 100 mg/mL streptomycin for more than 16 hours until subsequent experiments. Intracellular ROS generation caused by oxaliplatin in neuro2A cells was determined using CM-H_2_DCFDA. CM-H_2_DCFDA is converted by an intracellular esterase into 2’,7’-dichlorodihydrofluorescein (DCFH). The non-fluorescent DCFH is then oxidized by ROS to the highly fluorescent compound 2’,7’-dichlorofluorescein. The cells were incubated for 30 minutes with 10 μM CM-H_2_DCFDA at 37 °C, followed by treatment of drugs for 1 hour. Fluorescence intensity was measured using a microplate reader (Infinite M200; Tecan Japan Co., Ltd., Kanagawa, Japan) at 485 nm (excitation) /520 nm (emission).

### Statistics

Results are expressed as the means ± S.E.M. or means ± S.D. All statistical analyses were performed with StatLight2000 (Yukms Co., Ltd., Tokyo, Japan). The statistical significance of the difference between control groups and oxaliplatin-treated groups was calculated using Student’s *t* test. The behavioral data for GJG tests were analyzed by one-way analysis of variance and post hoc multiple comparisons using Dunnett’s test. A difference was considered significant at *P* < 0.05.

## Additional Information

**How to cite this article**: Kono, T. *et al.* Preventive effect of oral goshajinkigan on chronic oxaliplatin-induced hypoesthesia in rats. *Sci. Rep.* **5**, 16078; doi: 10.1038/srep16078 (2015).

## Supplementary Material

Supplementary Information

## Figures and Tables

**Table 1:** Influence of oxaliplatin (L-OHP) on peripheral blood flow and preventive effect of goshajinkigan (GJG) in rats.

	day 3	day 10	4 weeks		8 weeks	
			DW	GJG	DW	GJG
Head	108.3 ± 2.9	91.9 ± 4.5	89.9 ± 2.6*	87.9 ± 3.2	93.5 ± 3.8	93.1 ± 1.7
Right ear	101.5 ± 5.6	87.7 ± 2.0**	91.2 ± 3.4	91.8 ± 2.2	92.3 ± 4.1	91.4 ± 2.2
Left ear	104.8 ± 4.3	85.3 ± 2.2**	95.7 ± 2.9	95.0 ± 2.3	91.4 ± 3.2	91.5 ± 1.6
Right forelimb	100.8 ± 1.9	91.4 ± 3.1	89.0 ± 2.7**	101.7 ± 2.7^##^	100.3 ± 2.0	99.8 ± 1.9
Left forelimb	97.5 ± 3.1	93.1 ± 3.8	87.3 ± 2.8**	98.0 ± 3.3 ^#^	97.2 ± 1.5	97.5 ± 1.5
Right hindlimb	114.2 ± 2.1	86.5 ± 6.9	88.3 ± 5.1	108.5 ± 4.4^##^	102.3 ± 4.4	104.8 ± 5.4
Left hindlimb	110.2 ± 0.9	85.8 ± 5.0	82.0 ± 4.5**	109.0 ± 4.5^##^	95.7 ± 4.7	98.9 ± 4.3
Tail	98.8 ± 2.5	90.9 ± 3.4	100.1 ± 2.2	99.2 ± 1.8	96.3 ± 3.3	105.8 ± 5.0

Mean ± S.E.M. (%); N = 7–8. *P < 0.05, **P < 0.01 vs naïve rats, ^#^P < 0.05, ^##^P < 0.01 vs L-OHP + DW.

**Table 2:** Maximum plasma concentration of components derived from goshajinkigan (GJG) after oral administration of GJG in rats.

Crude drugs	Compound	C_max_ (ng/mL)	T_max_ (h)
processed Aconiti tuber	Benzoylaconine^a^	0.012 ± 0.002	0.50 ± 0.00
	Benzoylmesaconine^a^	0.041 ± 0.006	0.44 ± 0.13
	Benzoylhypaconine^a^	0.013 ± 0.001	0.50 ± 0.00
	14-Anisoylaconine^a^	0.009 ± 0.000	0.31 ± 0.13
Corni fructus	Loganin^b^	43.7 ± 25.7	0.58 ± 0.20
	Morroniside^b^	53.7 ± 26.9	0.67 ± 0.26
Rehmanniae radix	Catalpol^b^	282.3 ± 118.7	1.00 ± 0.55
Moutan coutex	Paeoniflorin^b^	9.1 ± 1.2	0.58 ± 0.20

Mean ± S.D.; ^a^N = 4, ^b^N = 6. C_max_: maximum plasma concentration. T_max_: time to reach maximum plasma concentration.
