# Supplementary material for: Preventive effect of oral goshajinkigan on chronic oxaliplatin-induced hypoesthesia in rats
Source: Sci Rep. 2015 Nov 6;5:16078. doi: 10.1038/srep16078 (PMC4635352; doi:10.1038/srep16078)
Supplement: Supplementary Information [file srep16078-s1.pdf]

Supplementary information

Preventive effect of oral goshajinkigan on chronic oxaliplatin-induced hypoesthesia in rats

Toru Kono[1,2*], Yasuyuki Suzuki[3], Keita Mizuno[3], Chika Miyagi[3], Yuji Omiya[3], Hitomi Sekine[3], Yasuharu Mizuhara[3], Kanako Miyano[4], Yoshio Kase[3], and Yasuhito Uezono[4]

Corresponding author: Toru Kono

Center for Clinical and Biomedical Research, Sapporo Higashi Tokushukai Hospital, 3-1, N-33, E-14, Higashi-ku, Sapporo 065-0033, Japan.

Tel.: +81-11-722-1110

E-mail address: kono@toru-kono.com

Supplementary Figure S1.

Glutathione and N-acetyl-L-cysteine attenuate ROS generation induced by oxaliplatin in neuro2A cells. Glutathione (GSH) or N-acetyl-L-cysteine (NAC) were concurrently administered with oxaliplatin (L-OHP; 100 μM) for 1 h, and ROS generation was measured using CM-H_2DCFDA. ** $P < 0.01$ compared with control group; ## $P < 0.01$ compared with oxaliplatin alone.

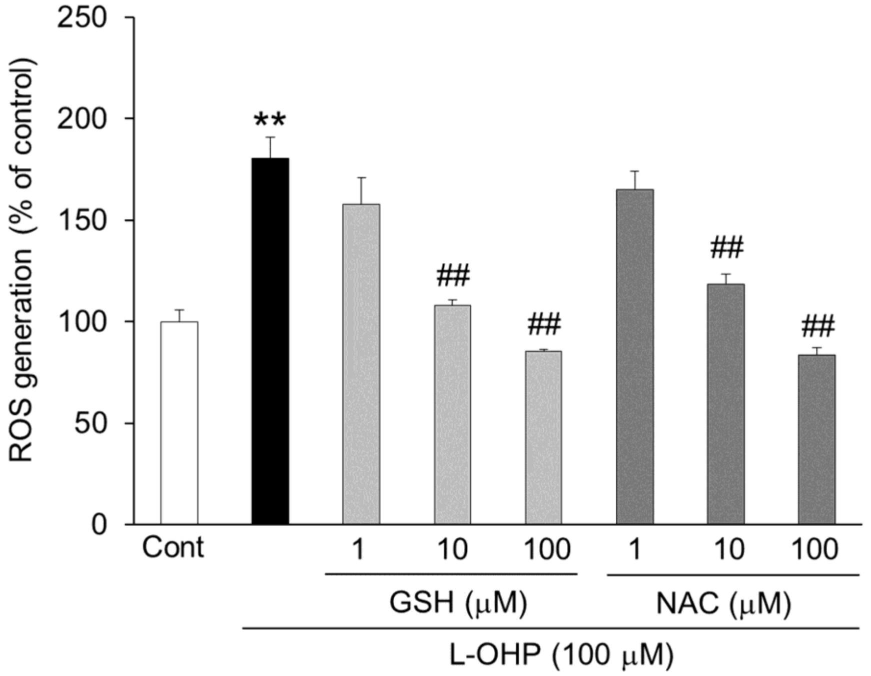

Supplementary method

Measurement of Morroniside by LC-MS/MS

Goshajinkigan (GJG) powder 500 mg (lot 2120107020) was suspended with 25 mL of methanol/purified water (50:50, v/v). After shaking and treating ultrasonication of the suspension for 15 min following by centrifugation at 3000 rpm, for 15 min, at 4°C, the supernatant was collected as an extract solution. After adding 25 mL of methanol/purified water (50:50, v/v) to the residue, re-extraction solution obtained by the same procedure described above was mixed to the first solution. The mixture (50 mL) was supplied to the LC-MS/MS analysis with a dilution step (×10000). Morroniside (MO) of GJG were measured by QTRAP®5500 system (AB SCIEX, Tokyo, Japan) equipped with an Agilent 1260 HPLC system (binary pump, online degasser, auto plate-sampler and column oven; Agilent Technologies, Tokyo, Japan). The columns used were Atlantis dC18 (2.1×100 mm, 3µm, Nihon Waters K.K., Tokyo, Japan) for MO analysis. The other conditions were as follows: flow rate, 0.2 ml/min; column temperature, 40°C; injection volume, 20 µL; mobile phase, solvent A(10 mmol/L ammonium acetate) - solvent B (acetonitrile containing 0.2 vol % acetic acid) (87/23,v/v); and ionization, electrospray ionization (ESI) with positive mode. MS/MS conditions for detection were summarized (see below). The low limits of the quantification were 0.101 ng/mL for MO. GJG (lot 2120107020) contains 0.425% of MO.

Optimized mass spectrometer acquisition parameters used for analyte quantitation

	MS/MS Condition			CUR	TEM	GS1	GS2	CAD	CE	DP	CXP	EP
Ion mode	Q1(*m/z*)		Q3(*m/z*)	(psi)	(℃)	(psi)	(psi)		(v)	(v)	(v)	(v)
Positive	424.150	$[M+NH_4]^+$	195.100	30	500	80	80	7	27	46	16	10

CUR, curtain gas ; TEM, temperature ; GS, ion source gas ; CAD, collision gas ; CE, collision energy ;
DP, declustering potential ; CXP,collision cell exit potential ; EP, entrance potential
